# Effect of case-specific 3D-printed models on surgical performance in cadaveric dissection—a randomized controlled trial

**DOI:** 10.1007/s00405-026-10099-x

**Published:** 2026-03-09

**Authors:** Adam Omari, Anders Nøhr, Elissa Suleiman, Per Cayé‑Thomasen, Mads Sølvsten Sørensen, Andreas Frithioff, Steven Arild Wuyts Andersen

**Affiliations:** 1 Copenhagen Hearing and Balance Center, Department of Otolaryngology–Head & Neck Surgery, Rigshospitalet, Copenhagen University Hospital, Copenhagen, Denmark; 2 Institute for Cellular and Molecular Medicine (ICMM), Faculty of Health and Medical Sciences, University of Copenhagen, Copenhagen, Denmark; 3 Center for HR & Education, Copenhagen Academy for Medical Education and Simulation (CAMES), RegionH, Copenhagen, Denmark; 4 Dept. of Clinical Medicine (ICM), Faculty of Health and Medical Sciences, University of Copenhagen, Copenhagen, Denmark

**Keywords:** Temporal bone, Mastoidectomy, Surgical training, 3D printing, Case-specific rehearsal, Simulation-based training

## Abstract

**Purpose:**

To investigate if training with case-specific 3D-printed temporal bone (TB) models improves trainees’ mastoidectomy performance in cadaveric dissection compared with training on a standard, generic-anatomy 3D-printed TB model.

**Methods:**

In this randomized controlled trial, 22 novice ORL residents performed a cadaveric mastoidectomy with posterior tympanotomy after training two procedures either on 3D-printed TB models of their specific cadaveric bone (CM, *n* = 11), or on 3D-printed TB with generic anatomy (GM, *n* = 11). Blinded experts assessed final-product performance using a modified Welling Scale. Secondary outcomes included correlation between performance on 3D-printed models and cadavers, questionnaires on model quality, and usefulness for planning.

**Results:**

On their first 3D-printed model, the CM group performed better than the GM group (13.1 vs. 10.9 points; *p* < 0.01). In their second 3D-printed procedure, scores were similar (15.8 vs. 15.7; *p* = 0.9) as both groups improved significantly from the first to the second procedure (*p* < 0.001). In subsequent cadaver dissection, the CM group scored lower than the GM group (13.2 vs. 14.4; *p* < 0.001). Correlation between performance on 3D-printed models and cadavers was not significant in the CM group (*r* = 0.45; *p* = 0.16) but very strong in the GM group (*r* = 0.77; *p* < 0.01). Questionnaire responses indicated high perceived realism and educational value in both groups.

**Conclusion:**

Training with case-specific 3D-printed TB models did not benefit or correlate with cadaveric mastoidectomy performance in novice residents. This supports the use of generic-anatomy, educational 3D-printed TB models in the early training stages and highlights the importance of aligning simulation fidelity with learner experience.

**Trial registration:**

Not applicable.

**Supplementary Information:**

The online version contains supplementary material available at 10.1007/s00405-026-10099-x.

## Introduction

Mastoidectomy is a core surgical procedure in otology, that grants access to the middle and inner ear [1, 2]. The surgeon must navigate a narrow and highly variable bony corridor in close proximity to critical structures where errors can lead to complications [1, 3]. Simulation-based training has become integral to contemporary training curricula to ensure basic skills acquisition in a risk-free learning environment. The most realistic simulation modality remains cadaveric temporal bone (TB) dissection [4] but increasing costs, limited supply, and logistical hurdles have driven educational programs towards other simulation modalities [5, 6]. Among these, 3D-printed temporal bone (TB) models have become widely accessible and increasingly realistic owing to continuous advances in 3D-printer resolution, material science, and cost-effectiveness [7]. 

Training on 3D-printed TB models improves mastoidectomy final-product performance [8–12] and their implementation into training curricula is generally supported by both educators and trainees [13]. Skills acquired on 3D-printed TB models transfer to cadaver dissection [10, 11] but existing training models rely on generic-anatomy models, representing well-aerated TBs with normal anatomy. However, this does not reflect the conditions when training on cadaveric TBs or real-life surgery. On one hand, training model standardization allows educators and researchers to homogenize training and assessment by removing anatomical variability. On the other hand, this approach might leave trainees naïve to the naturally occurring anatomical variation inherent to cadaver training and real-life surgery. Bridging this gap in performance between educational 3D-printed models and more realistic conditions such as training on cadaveric specimens requires attention to the *instructional design*—the specific tools and strategies used to best plan and implement training [14, 15]. 

Outside the educational use of TB models for training, case-specific models based on clinical imaging of the patient allow the surgeons to rehearse and plan on patient-specific anatomy before performing the surgery in real-life. This would potentially aid in preparing for specific anatomical variations such as the facial nerve course, tegmen height, sigmoid sinus position, pneumatization, and sclerotic patterns. This is proposed to have benefits for the patients [16–18] even though higher-level evidence remains limited [19]. Nonetheless, deliberate practice on case-specific 3D-printed TB models aligns with compelling instructional design features [15] and may be hypothesized to improve skills transfer in simulation-based training.

No studies on 3D-printed TB models have explored the use of case-specific TB models for training. It was until recently very resource intensive to manufacture such models given the manual effort involved, perhaps explaining why their use was restricted to select clinical cases [19]. Yet, ongoing technological advances make broader adoption more feasible by replacing manual processing steps with automated solutions using thresholding [16] or neural networks [20] to more efficiently transform imaging into 3D-printable models.

In this study, we aimed to investigate the effect of training on case-specific versus generic-anatomy 3D-printed temporal bone models on novice trainees’ mastoidectomy performance and transfer of skills to subsequent cadaveric dissection. We secondarily explored the correlation between training performance and cadaver performance, and collected the trainees’ perceptions of the models’ value in simulation-based training and for surgical planning. The overarching goal is to establish the role of case-specific models in simulation-based training of mastoidectomy for novice trainees.

## Methods

### Study design

This was a randomized, controlled trial (RCT) of an educational intervention for otorhinolaryngology (ORL) residents.

### Participants and setting

**Fig. 1:**
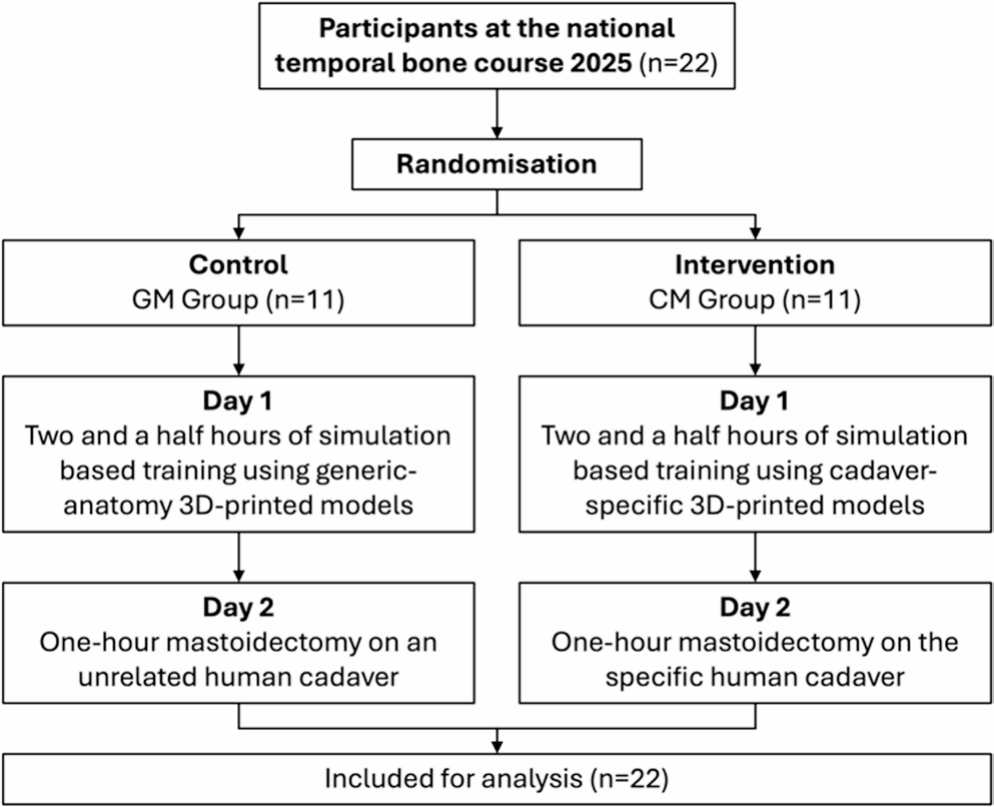
Study flow diagram. For all participants, the training on Day 1 was preceded by a day with theoretical lectures on the ear anatomy and surgical approaches to the middle and inner ear

**Fig. 2:**
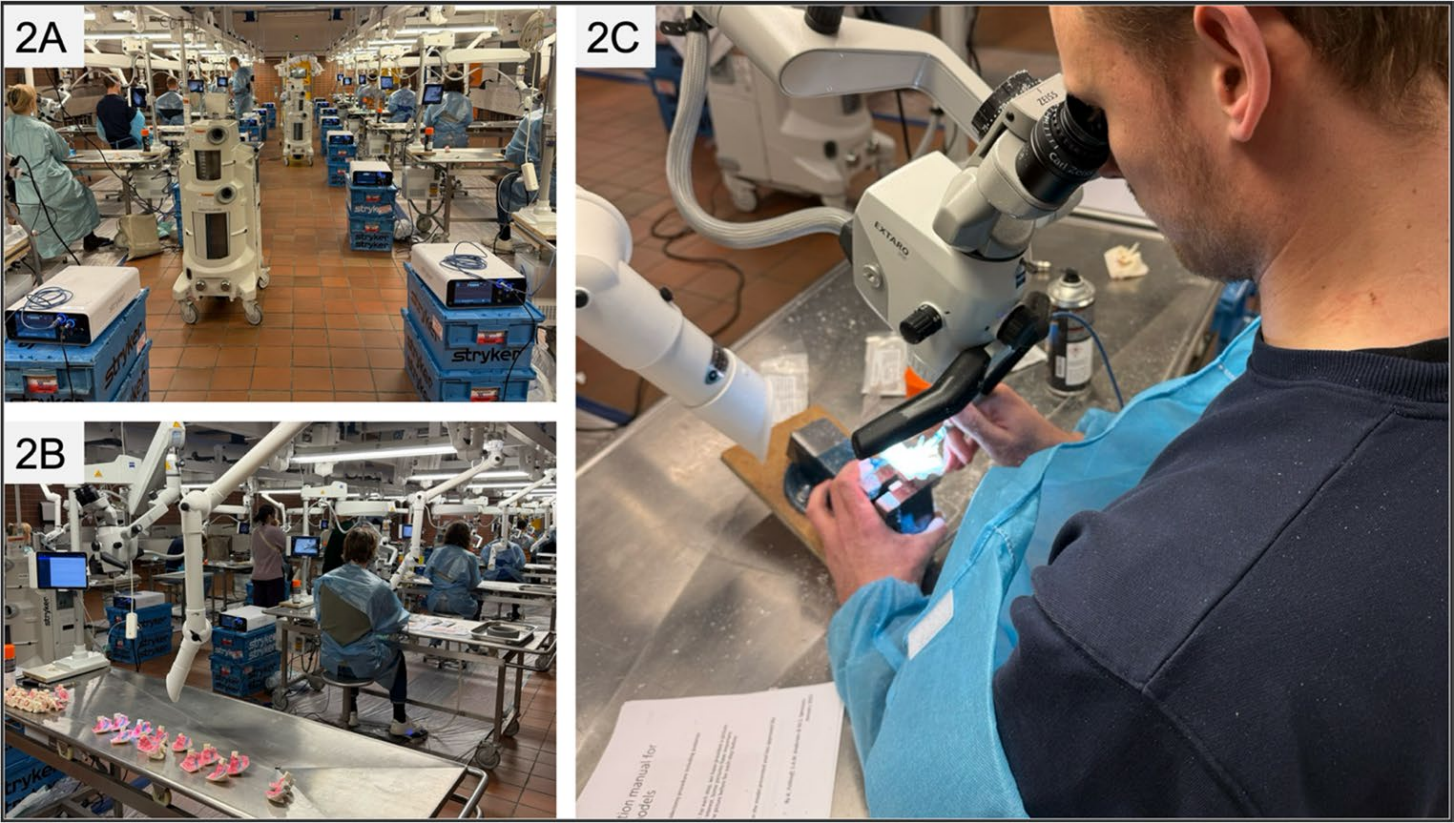
Simulation training environment. All participants drilled the 3D-printed models and the cadavers in the same environment in their separate training station (2A and 2B). Each training station was equipped with a conventional otosurgical drill, a standard selection of drill bits, otomicroscope and dissection manual. The 3D-printed models were fixed in a vice for stability during drilling (2C) and cadaver heads were fixed in custom mount (not shown)

22 ORL residents (PGY2-4) were invited and enrolled in relation to the compulsory annual Danish national cadaver temporal bone dissection course at Rigshospitalet, Copenhagen, in January 2025. Residents completed two mastoidectomy procedures on 3D-printed TB models; the following day they performed the cadaveric dissection (Fig. 1). Both activities took place in the same dissection hall using identical drilling stations and microscopes (Fig. 2). Prior to hands-on training, all trainees received lectures on ear anatomy and surgical approaches to the middle and inner ear. Participants signed an informed consent form and completed a background questionnaire (Table 1).

**Table 1:** Participant characteristics

Age, years (mean ± SD)	Overall (*n* = 22)	GM Group (*n* = 11)	CM Group (*n* = 11)
	34.1 (2.8)	35.5 (2.7)	32.6 (2.1)
Sex: male/female (% male)	17/5 (77%)	8/3 (73%)	9/2 (82%)
ENT experience, years (mean ± SD)	3.8 (1.2)	3.9 (1.5)	3.6 (0.8)
Overall surgical experience, years (mean ± SD)	4.1 (1.5)	4.5 (1.8)	3.8 (1.0)
Previous TB course participation	0 (0%)	0 (0%)	0 (0%)
Previous VR/3D-print TB simulation training	3 (14%)	2 (18%)	1 (9%)

VR: Virtual Reality. SD: Standard deviation. GM: Generic-anatomy Models. CM: Case-specific Models

### Randomization

Participants were randomized 1:1, to training on either (1) two identical generic-anatomy 3D-printed TB models (Generic-anatomy Model (GM) group, control), or (2) two identical case-specific 3D-printed TB models (Case-specific Model (CM) group, intervention) (Fig. 1). For training on 3D-printed models, participants were randomized to drilling stations pre-designated for either the GM or CM group (Supplementary Material 1). Group allocation was revealed prior to drilling at the drilling stations to align with typical use in clinical conditions. For cadaver dissection the following day, each cadaver head was shared between a participant from each group to balance anatomical variation difficulty.

### Intervention

Participants in both groups had 2½ hours to complete two anatomical mastoidectomies on 3D-printed models. They progressed to the second model at their own pace or after reaching the halfway point of the session, to ensure adequate time for their second mastoidectomy. Instructors provided no hands-on assistance and answered only general questions, such as those concerning correct use of the surgical equipment. Participants had access to a printed dissection manual with step-by-step instructions and corresponding images along with a perfectly drilled generic-anatomy model available upon request. Otosurgical drills (S2 πDrive Drill System, Stryker, Kalamazoo, USA) with an array of drill bits and an operating microscope (EXTARO 300, Zeiss Group, Oberkochen, Germany) were provided for each participant. Suction and irrigation were not necessary as plastic drill dust is best cleared with compressed air.

The day after training on 3D-printed models, each participant performed an anatomical mastoidectomy on a human cadaver with a time limit of one hour. This duration was a course constraint and based on historical course data on cadaver mastoidectomy. Trainees worked in pairs on each head: one participant drilled the right side while the partner assisted; roles were swapped once the head was flipped to the contralateral side. All microscope feeds were displayed for the assistant and recorded to control for a possible observational advantage of watching before drilling.

### 3D-printed temporal bone model

To acquire case-specific 3D-printed temporal-bone models for the CM group, one randomized temporal bone on each cadaver was imaged in the weeks before the course on a cone-beam CT (CBCT) scanner (3D Accuitomo 170; J. Morita Mfg. Corp., Kyoto, Japan). Scans were obtained with a clinical high-resolution protocol (voxel size 0.08 mm, FOV 60 × 60 mm, 360° rotation, 30 s exposure, 80 kV, 6 mA). The cadaveric temporal bones were screened to exclude prior surgery or gross ear pathology. While natural anatomical variations (e.g., pneumatization, tegmen height, sigmoid sinus position) were present, no malformations or severe sclerosis were observed. The resulting DICOM data were imported into the VESTool (in-house software for 3D segmentation), which is based on the virtual environment from our Visual Ear Simulator) [21]. VESTool was used to first resample the virtual models into 0.2 mm voxel size and then segment the bony volume based on a combination of interactive and localized thresholding features with minimal manual effort required. The virtual models were finally exported as standard tessellation language (STL) files to undergo 3D-printing. Conversely, the generic-anatomy 3D-printed temporal bone models offered to the GM group relied on our previously described CBCT data set “*Delta”*, which is directly 3D-printable [22]. A cube for fixing the 3D-printed models to a holder was added to all models using Blender v4.0 (Blender Foundation, Amsterdam, Netherlands). All models underwent 3D-printing with identical specifications (Supplementary Material 2). Given the unique nature of all case-specific models, they were individually drilled by the author (AO) on a test bench to validate accuracy against the CT imaging data and quality control for errors such as verifying that the simulated facial nerve could be inserted past the second genu and into the horizontal part of the fallopian canal.

### Outcome

**Fig. 3:**
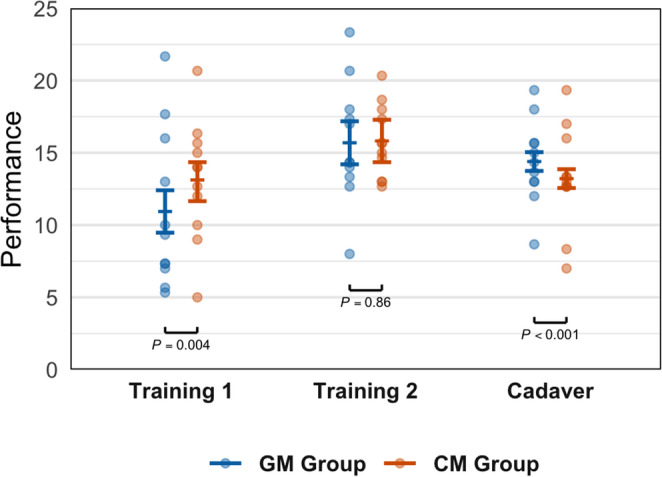
Final-product performance in training and transfer. Lines and whiskers show LMM-adjusted mean final-product performance scores on the modified welling scale (WS) with 95 % confidence intervals. Dots represent each participant’s unadjusted WS scores averaged across the three raters. P values calculated using unpaired Welch’s t-test. GM = Generic-anatomy models (n = 11); CM = Case-specific models (n = 11)

The primary outcome was mastoidectomy performances assessed using the modified Welling Scale (WS), a binary grading instrument for final-product analysis [23]. The chorda tympani was not present in the 3D-printed models reducing the maximal score from 26 to 25 points. Three blinded experts rated the final products of each cadaver dissection (Fig. 3). The 3D-printed models were anonymized and shuffled before rating by the same three experts.

Secondary outcomes include a questionnaire on planning and preparedness (Table 2). This was based on existing questionnaires [24, 25] but simplified to fit the context of our study with participants being novice trainees. We further included the modified Mowry’s questionnaire on the usefulness of the 3D-printed models compared to human cadavers [26]. The questionnaire includes questions on bone density, landmark fidelity, drilling feel, middle‑ear detail, and overall educational utility (Table 3).

**Table 2:** Planning and preparation questionnaire

Item	GM Group (*n* = 11)	CM Group (*n* = 11)	*p*-value
1. The model increased my confidence in performing cadaver drilling	5.0 [4.0–5.0]	4.0 [4.0–5.0]	0.74
2. The model made me able to better identify anatomical landmarks in the cadaver	4.0 [3.5–5.0]	4.0 [4.0–4.0]	0.97
3. The model helped me to understand the spatial relationships	4.0 [3.5–5.0]	4.0 [4.0–4.5]	0.92
4. The model helped me recall critical steps during the cadaver dissection	4.0 [3.5–5.0]	4.0 [3.5–4.5]	0.81
5. The model made me sufficiently prepared to perform the cadaver drilling	5.0 [3.0–5.0]	4.0 [4.0–5.0]	0.75
6. Drilling the model was useful for pre-surgical planning and rehearsal	4.0 [4.0–5.0]	4.0 [4.0–5.0]	0.86
7. Drilling a case-specific model motivated me to spend more time to remember the specific anatomy	n/a	3.0 [2.0–3.5]	–
8. Before my first mastoidectomy on a patient, I would like to dissect a patient-specific model	n/a	4.5 [4.0–5.0]	–
9. I would have liked to inspect my case-specific model during the cadaver drilling	n/a	4.0 [4.0–5.0]	–

Scored on a Likert scale of one (lowest) to five (highest). Values shown as median [IQR]. IQR = interquartile range (25th–75th percentile). p‑values from two‑sided Mann–Whitney U tests. GM: Generic-anatomy Models. CM: Case-specific Models. Question 7–9 related to the case-specific models and were therefore only offered to the CM group

**Table 3:** Mowry questionnaire

Questionnaire item	GM Group (*n* = 11)	CM Group (*n* = 11)	*p*‑value	Adj *p* (Bonferroni)
1. External contour	3.0 [2.5–4.0]	3.0 [3.0–4.0]	0.48	1
2. Texture of drilled plastic mastoid architecture	2.0 [1.5–3.0]	3.0 [2.0–3.0]	0.39	1
3. Anatomy of the antrum (incus pointer)	3.0 [2.5–3.0]	3.0 [2.0–4.0]	0.85	1
4. Tegmen contour	3.0 [2.0–3.0]	3.0 [2.5–4.0]	0.48	1
5. Otic capsule contour and density	3.0 [2.8–3.0]	3.0 [2.5–3.0]	0.72	1
6. Sigmoid sinus contour	3.0 [2.0–4.0]	3.0 [2.0–3.5]	0.25	1
7. Change‑in‑pitch realism	2.0 [2.0–3.0]	2.0 [1.5–3.5]	0.82	1
8. Reflectivity of the model	2.0 [2.0–3.0]	3.0 [2.0–3.5]	0.12	1
9. Absence of odour when dissected	2.0 [2.0–3.0]	2.0 [2.0–3.0]	0.51	1
10. Dust formation during dissection	3.0 [2.0–3.0]	3.0 [2.0–3.0]	0.75	1
11. 3D‑print support material absent	2.0 [1.5–2.5]	3.0 [2.0–3.0]	0.077	1
12. Facial nerve	3.0 [2.0–3.0]	3.0 [2.5–3.0]	0.48	1
13. Facial nerve recess	2.0 [2.0–2.0]	3.0 [3.0–4.0]	**0.048**	**0.72**
14. Overall anatomical fidelity	3.0 [3.0–3.0]	3.0 [3.0–3.5]	0.79	1
15. Suitability as a simulator	4.0 [3.0–5.0]	4.0 [4.0–4.0]	0.92	1
**Total Mowry score**	41 [37–47]	44 [42–46]	0.22	1

The Mowry Questionnaire [26] was distributed to all participants after cadaver dissection on Day 2. Values shown as median [IQR]. IQR = interquartile range (25th–75th percentile). For Total Mowry Score, the individual totals were computed by summing only answers with complete responses (*n* = 7 in each group) as responses with missing items were discarded for analysis. Scores are based on the Mowry Questionnaire. p‑values were calculated using two‑sided Mann–Whitney U tests

### Sample size

The sample size was a convenience sample. Every ORL resident who attended the annual national TB course was invited and consented to participate. The sample size satisfied the minimum recommendations for normality assumptions of the central limit theorem commonly used in educational research.

### Statistical methods

Statistical analyses were carried out in SPSS version 27 (IBM, New York, USA). Because each procedure was evaluated by multiple raters and each trainee contributed multiple performances, we applied linear mixed-effects models (LMMs) as recommended by Leppink [27]. For cadaveric dissection scores, the LMM included group (GM vs. CM), rater, and cadaver ID as fixed factors. Observational advantage from watching the partner drill the contralateral TB was not significant (*p* = 0.51) and was excluded in the model. A significant interaction between training group and cadaver ID was detected and retained in the model. The LMM for 3D-printed model performance included training group, rater, and procedure number as fixed factors. No interactions were found.

Survey scores were compared between groups with the two-tailed Mann–Whitney U test. For the modified Mowry questionnaire, only the total score was a pre‑specified secondary outcome, and item‑level analyses were exploratory and reported with both unadjusted and Bonferroni adjusted p‑values. Pearson’s *r* was used for correlation analysis between cadaveric and 3D-printed model performances with Fisher’s *z* transformation applied for 95% confidence intervals (CI). The coefficient of determination (*R²*), representing the proportion of variance explained, was calculated by squaring *r.* P values < 0.05 were considered statistically significant.

## Results

Both groups had comparable baseline characteristics including age, sex, ORL experience, and previous training (Table 1).

### Training performance

In their first training performance on 3D-printed TB models, the mean final product performance score was higher for the CM group with 13.1 points (95% CI [11.7–14.6]) compared with the GM group with 10.9 points (95% CI [9.5–12.4]) (LMM, *P* = 0.004). In the second procedure, the groups had reached almost identical performance with the CM group scoring 15.8 points (95% CI [14.4–17.3]) and the GM group scoring 15.7 points (95% CI [14.2–17.2]) (LMM, *P* = 0.86). Performance increased significantly from the first to the second procedure for both the CM and the GM group (LMM, *P* < 0.001) (Fig. 3).

### Cadaver performance

For subsequent cadaver dissection performance, mean final product performance scores were 13.2 points (95% CI 12.6–13.9) for the CM group and 14.4 points (95% CI 13.7–15.0) for the GM group. This meant that the CM group demonstrated 1.2 points (≈ 9%) lower performance scores than the GM group (LMM, *P* < 0.001), favoring training using generic-anatomy models instead of case-specific models (Fig. 3). We observed a significant group-cadaver interaction in the LMM analysis, indicating heterogeneity of the GM–CM difference across cadavers. This could reflect anatomy-specific effects, but anatomical difficulty of each specimen was not formally scored. Finally, there was wide variation in performance among trainees throughout training and cadaver performance (Fig. 3) even though the study population was relatively homogenous (Table 1).

### Correlation between training and cadaver performance

In the CM group, the correlation between the last training procedure and cadaver performance was moderate yet not significant (*r* = 0.45; *p* = 0.16; 95% CI [− 0.20 to 0.83]). This corresponds to *R*^*2*^ *= 21%*, indicating that training performance explained about 21% of the variability in cadaver scores. Conversely, the correlation in the GM group was very strong (*r* = 0.77; *p* = 0.005; 95% CI [0.32–0.94]), explaining about 60% of the variance in cadaver performance (*R*^*2*^ = 60%).

### Questionnaire results

The self-reported planning and preparedness from rehearsing on 3D-printed models were high and at a similar level between groups. In the case-specific questions, the majority in the CM group welcomed training on a case‑specific model before their first real patient surgery and would also prefer to keep the drilled model as an intra-operative reference (Table 2). The median modified Mowry questionnaire scores were 44 points (IQR: 42–46 points) for the CM group which was relatively similar to the GM group with median 41 points (IQR: 37–47 points) (*p* = 0.22). Item 13 (facial nerve recess realism to cadaver) was significantly higher in the case-specific group (*p* = 0.048) but this did not remain significant after Bonferroni correction (adjusted *p* = 0.72). No other items reached significance (Table 3).

## Discussion

This randomized trial is the first to investigate the effect on cadaveric mastoidectomy performance after training using case‑specific 3D‑printed TB model compared with training on a generic-anatomy, educational 3D-printed TB model. We found that novice trainees who trained using case‑specific models scored lower in subsequent cadaveric mastoidectomy (i.e., poorer transfer of skills) compared with peers who trained on a generic anatomy TB model, thereby disproving the hypothesis that case-specific training translates to better cadaveric dissection performance of novice trainees. Further, 3D-model performance scores for the CM group showed only a moderate, non-significant correlation with cadaver performance, whereas scores in the GM group demonstrated a very strong and significant correlation. Subjectively, trainees rated both GM and CM models highly in realism and usefulness for planning and preparedness, with majority in the CM group stating they would prefer to train on a case‑specific model before their first live operation. Models in both groups surpassed the 40-point model usefulness threshold reported by Mowry et al. [26] which relied on a panel of expert surgeons assessing the similarity between model and cadaveric dissection [26]. 

Prior studies limited to educational 3D-printed TB models have shown clear transfer gains to cadaveric dissection: one study found that a single training procedure increased subsequent cadaver scores by about four points compared with no training [11], and three repeated procedures on 3D-printed models were found to result in a 2% improvement in performance compared with time-matched virtual reality (VR) simulator training [10]. We also found mean final product performance scores rose between the first and second procedure in both groups, indicating a learning curve by repeated practice similar to real supervised surgery [28]. This contrasts findings in a prior study that reported no score improvements over three repeated procedures [10]. A possible explanation could be time allotted per training procedure (90 min in our study vs. 60 min previously), or choice of model material where slightly translucent materials—such as the ABS we used—offer vital visual drilling cues and boosts performance compared with opaque materials [12]. Our results also indicate that simply adding training procedures improves skills, suggesting that the learning potential of one modality should be exhausted before learners progress to further training using other modalities or supervised surgery.

Case-specific training has been studied in VR temporal bone surgical simulation and shows mixed educational value. While it has become feasible to create simulations based on clinical image data [29], they may still be limited by lack of realistic tactile and visual cues of key anatomic structures found in generic-anatomy simulators, which can lead to unexpected effects where for example trainees outperform attending surgeons in simulated TB drilling for cochlear implantation [30]. Still, a heterogenous group of trainees rated a 90-minute case-specific VR training highly for planning, training, and confidence [31], and similar benefits were observed when trainees were asked to rate case-specific VR models after drilling the corresponding cadaver [32]. Another study showed confidence after case-specific VR training correlated with cadaver performance, but gains depended on experience and task difficulty. Specifically in novice trainees, confidence barely improved for intermediate or advanced drilling tasks as opposed to greater gains reported for more experienced trainees [33]. The only RCT of case-specific vs. generic VR TB surgical training also found no benefit on the transfer of skills to 3D-printed TB model performance of novices [34]. Thus, positive sentiment does not translate into objective gains for novice trainees and ultimately the limited benefit of case-specific training for novices appears to span across simulation-based TB training modalities.

In our study, the GM–CM difference varied across cadavers, as shown by a significant group-cadaver interaction. This suggests that case anatomy may moderate the effect of case-specific training. The cadaver temporal bones displayed natural anatomical variation but we did not grade difficulty or select specimens based on pathology. Case-specific training may therefore still be beneficial when anatomy deviates markedly from generic anatomy of the educational model, for example in cases of malformations, cholesteatoma, low tegmen, sclerotic mastoid, or small temporal bones. Clinical reports in experienced hands align with this and show benefits mainly in anatomically difficult or pathological cases [17, 18]. However, the literature may overrepresent complex cases because surgeons seldom study or publish on simple and straightforward cases.

Case-specific TB models have higher physical resemblance than generic-anatomy model but this might only benefit trainees of a certain level of experience [35] and warrants careful consideration of educational need of the trainee [36]. Instead, the functional task alignment (i.e., does training actually train the skills required for the real task of the simulation) appears to be more important than physical resemblance for optimizing training outcomes. It might be speculated, that fine case-specific details may distract novice trainees from focusing on basic learning objectives such as drill control, landmarks, and basic steps. Our results therefore inform educators that generic-anatomy models should be preferred for novices given better alignment with their foundational learning needs.

Manufacturing case‑specific models requires considerably more resources and effort than merely printing standard TB models, making the latter more cost-effective [37]. To create a case-specific model, the multi-step pipeline [19] currently involves image acquisition such as CT or CBCT imaging, segmentation using semi-automated processes, manual correction of errors, and careful quality control of printed models. These steps are time consuming with 1–4 h spent per model depending on need for repeated adjustments and iterations, and consequently far more costly than 3D-print filament costs which in our case is about USD ~ 2 per model and using a 1,250 USD consumer-grade FDM printer. Routine case-specific 3D-printing remains unlikely to be cost-effective for initial training until automation can reduce the time consuming manual steps to a minimum [19]. For more experienced trainees who are transitioning from simulation-based training to actual patients, there might be sufficient value in case-specific training to justify the costs. Nonetheless, this remains speculative until dedicated studies have addressed this question.

A key strength of this study is its randomized design and that it follows recommendations for how instructional designs of simulation-based training should be compared [15] with transfer to other settings [36]. Three blinded experts rated performance on a validated final-product instrument (WS), adding objective assessment beyond the face/content validity commonly reported in the literature [13]. To our knowledge, this is also the first study to quantify the correlation between case-specific TB training performance and cadaveric performance in any simulation modality. This study also has several limitations. First, our cohort contained relatively few trainees but enough to justify the use of parametric statistics also due to repeated performances by the participants. Also, we standardized training to two models per trainee to allow closer comparison with prior studies on 3D-printed TB models. Additional practice beyond two procedures might have affected results, and next-day cadaver transfer created a brief washout period that may have reduced some immediate priming effects but on the other hand allows for time-dependent consolidation of technical skills [38]. 

In a progressive simulation-based training curriculum, simulator fidelity should be calibrated to learner stage [39]. Future work should therefore explore the effect of case-specific training in trainees who have advanced beyond foundational skills in direct comparison with generic-anatomy models using objective assessment. This is supported by benefits in experienced trainees compared to novice trainees in VR simulation [33], and also aligns with more general recommendations to explore instructional design principles to find *what* works, *when* and for *whom* to optimize learning effectiveness in simulation-based training [14, 15]. Future studies should also test defined pathological cases to determine whether pathology-specific training could confer an advantage over natural variation in healthy TBs. Finally, our findings of wide variation in individual final-products scores emphasize the need for personalized approaches such as mastery-learning programs [40, 41]. Mastery-learning programs advance trainees based on demonstrated proficiency rather than on training duration or number of training attempts [41, 42]. 

## Conclusion

For novice surgeons learning mastoidectomy and posterior tympanotomy, training on case-specific 3D-printed temporal bones resulted in lower cadaveric performance compared with training on comparable models designed specifically with education in mind. Performance in case-specific training also demonstrated weaker correlation with cadaver performance, despite trainees’ favourable perceptions regarding realism and value for surgical planning. These findings, together with the higher manufacturing costs, caution against the routine use of case-specific models in early training and underline the importance of aligning simulator fidelity with learner stage to ensure effective simulation-based training.

## Supplementary Information

Below is the link to the electronic supplementary material.

Supplementary Material 1 (DOCX 16.8 KB)

Supplementary Material 2 (DOCX 15.7 KB)
