# Supplementary material for: Effect of case-specific 3D-printed models on surgical performance in cadaveric dissection—a randomized controlled trial
Source: Eur Arch Otorhinolaryngol. 2026 Mar 9;283(6):3671–9. doi: 10.1007/s00405-026-10099-x (PMC13249690; doi:10.1007/s00405-026-10099-x)
Supplement: Supplementary file 1 — Supplementary Material 1 (DOCX 16.8 KB) [file 405_2026_10099_MOESM1_ESM.docx]

**Electronic Supplementary Material 1: Organization of 3D-printed TB training**

The trainees will be doing the cadaver surgery in pairs of two, where each trainee will perform the cadaver surgery on either the left or right side of the cadaver heads. The 12 pairs will be randomized into a control and an intervention group and then assigned numbers 1-12. For example, the two trainees in pair number 1 will be called 1a and 1b and they will perform mastoidectomy on cadaver head number 1. This was done to minimize the influence of two potential confounders: Firstly, some cadaver heads are more difficult than others to perform correct surgery on. Secondly, having lectures *before* simulation could be a potential advantage (or vice versa). Thus, Trainee “a” in each group will have lectures *before* the simulator practice. Trainee “b” in each group will have lectures *after* the simulator practice.

a = lectures before simulation *italic = control group (GM Group)*

b = lectures after simulation **bold = intervention group (CM Group)**

1^st^ pair: *1a* + **1b** Cadaver head 1

2^nd^ pair: **2a** + *2b* Cadaver head 2

3^rd^ pair: *3a* + **3b** Cadaver head 3

4^th^ pair: **4a** + *4b* Cadaver head 4

5^th^ pair: *5a* + **5b**  Cadaver head 5

6^th^ pair: **6a** + *6b* Cadaver head 6

7^th^ pair: *7a* + **7b**  Cadaver head 7

8^th^ pair: **8a** + *8b* Cadaver head 8

9^th^ pair: *9a* + **9b** Cadaver head 9

10^th^ pair: **10a** + *10b*  Cadaver head 10

11^th^ pair: 11a + **11b** Cadaver head 11

|  | Lecture before simulator practice | Lecture after simulator practice |
| --- | --- | --- |
| ***Control***  ***(GM group)*** | *Trainee:*  *1a-3a-5a-7a-9a-11a* |  |
|  |  | *Trainee:  2b-4b-6b-8b-10b* |
| **Intervention (CM group)** | **Trainee: 2a-4a-6a-8a-10a** |  |
|  |  | **Trainee: 1b-3b-5b-7b-9b-11b** |
