# Supplementary material for: Effect of case-specific 3D-printed models on surgical performance in cadaveric dissection—a randomized controlled trial
Source: Eur Arch Otorhinolaryngol. 2026 Mar 9;283(6):3671–9. doi: 10.1007/s00405-026-10099-x (PMC13249690; doi:10.1007/s00405-026-10099-x)
Supplement: Supplementary file 2 — Supplementary Material 2 (DOCX 15.7 KB) [file 405_2026_10099_MOESM2_ESM.docx]

**Electronic Supplementary Material 2: 3D-print specifications**

|  | 3D-Print Specifications |
| --- | --- |
| All 3D-printed models | All models for offered to the GM and CM group were imported into Bambu Studio v 1.10.1and then printed in-house using a fused deposition modeling (FDM) 3D-printer (X1-Carbon, Bambu Lab, Shenzhen, China).  3D-printer settings included default nozzle (0.4mm), 0.2mm slice thickness, infill of 85 %, variable width wall generator (Arachne mode) and printed in a cranio-to-caudal orientation. All other settings were default. The models were 3D-printed in a “natural”-colored, slightly translucent, ABS (acrylonitrile butadiene styrene) filament. Print time was 3-4 hours, and approximate filament costs were ~2 USD/model.  Post-processing consisted of removing scaffolds, coloring the sigmoid sinus and dura using acrylic paint, and insertion of a colored wire into the fallopian canal to represent the facial nerve. When jagged edges impeded insertion of the wire, we applied Gaussian smoothing to the canal wall in the VESTool software and re-printed the model with identical print settings to ensure all models had a simulated facial nerve. |
